# Airway Challenges in Maxillofacial Trauma: A Retrospective Study of 100 Cases

**DOI:** 10.7759/cureus.81470

**Published:** 2025-03-30

**Authors:** Afnan Amjad, Nusrat Shaheen

**Affiliations:** 1 Anesthesia and Critical Care, Lady Reading Hospital Medical Teaching Institution, Peshawar, PAK; 2 Anesthesia and Intensive Care, National Maternity Hospital, Dublin, IRL

**Keywords:** airway management, anesthetic management, direct laryngoscopy, fracture types, maxillofacial trauma, nasal intubation, videolaryngoscopy

## Abstract

Introduction: Maxillofacial trauma creates uniquely challenging airways. Anesthesiologists often struggle with initial access, maintaining the airway during surgery, safe extubation, and postoperative monitoring. These cases require experience and careful planning.

Methods: This retrospective analysis included 100 patients who were presented with maxillofacial trauma. Patients were assessed for airway patency based on preoperative criteria, including Mallampati classification, thyromental distance, mentohyoid distance, and neck movement. The intubation technique was selected based on fracture type and the anticipated difficulty in securing the airway. Techniques used included direct laryngoscopy, videolaryngoscopy, and blind nasal intubation.

Results: Direct laryngoscopy was the most commonly used technique in 53% of patients with minimal facial trauma. However, videolaryngoscopy was used in 23% of cases where a difficult airway was anticipated, while blind nasal intubation was performed in 24% of patients. The most common fractures were mandibular (29%), followed by nasal (24%) and maxillary (24%).

Conclusion: Airway management in maxillofacial trauma patients requires a tailored approach, with videolaryngoscopy being a valuable tool for difficult intubations. Proper preoperative evaluation and the collaboration between anesthesiologists and surgeons are essential for optimizing patient outcomes.

## Introduction

The maxillofacial region is made up of three main areas: the upper face, which includes the frontal region; the midface, which includes the maxilla, nasal complex, and zygomatic; and the lower face, embodied by the mandible. Maxillofacial fractures represent a prevalent category of trauma encountered in trauma centers globally [1]. The frequency of occurrence is reported to differ significantly across countries and regions within countries, due to variations in socioeconomic, demographic, and environmental factors, with prevalence rates fluctuating between 17% and 69%. It has been observed that the male gender dominates in as much as 85% of maxillofacial injuries worldwide [2-4].

Patients experiencing maxillofacial trauma pose major difficulties for anesthesiologists, as airway management in such instances can be complicated by altered anatomical structures [5]. The consequences of this condition vary from noteworthy functional and aesthetic difficulties to potentially life-threatening circumstances. Maxillofacial injuries can include deep lacerations, facial trauma, and potential injuries to the head, chest, and cervical spine. They may also be restricted to surface-level lacerations [6-8]. Traumatic displacement can lead to difficulties with ventilation or result in total obstruction of the airway. A heightened level of vigilance is essential for identifying displaced teeth within the airway of patients presenting with missing and fractured teeth, particularly in situations characterized by inadequate ventilation or low oxygen saturation despite a stable upper airway [9]. In these patients, the primary focus is on maintaining the airway while ensuring cervical spine stability, following the principles of Advanced Trauma Life Support when dealing with life-threatening injuries [10,11]. The primary objective of this study is to evaluate different airway management techniques in relation to maxillofacial fracture patterns, with particular focus on how preoperative airway assessments influence the selection and success of these techniques in the trauma setting.

Effective and secure airway management is crucial during the reconstruction and fixation of fractures. The morbidity and mortality rates in in-hospital trauma patients frequently stem from critical care errors involving airway and respiratory management. Airway management in maxillofacial trauma poses considerable challenges owing to the intricate anatomy and the risk of both internal and external airway obstruction. Accurately assessing the patient's airway, recognizing injury patterns, and demonstrating competence in advanced airway techniques are essential for effective management.

## Materials and methods

The study utilized a retrospective design, analyzing the medical records of 100 patients who presented with maxillofacial trauma over a six-month period. The study was conducted at Lady Reading Hospital, Peshawar, in the Maxillofacial Surgical Operation Theatre between July 2024 and January 2025. All patients were evaluated and treated for their injuries at a tertiary care center. Patients aged five years and above, who sustained injuries from road traffic accidents, falls, interpersonal violence, or sports incidents, and had complete medical records, were included in the study. Patients with incomplete medical records, pre-existing craniofacial anomalies, or injuries requiring immediate life-saving interventions that rendered standard airway assessment impossible were excluded. A thorough preoperative evaluation was conducted for each patient, which included a detailed assessment of airway patency using established criteria such as Mallampati classification, thyromental distance, and mentohyoid distance. In cases where facial fractures impacted airway accessibility, nasal patency was also assessed.

The anesthesia team carried out all intubations under general anesthesia, with techniques chosen based on the patient's fracture type and the anticipated difficulty in airway management. Direct laryngoscopy was used as the first-line approach for patients with adequate mouth opening and minimal facial trauma. In cases where access to the airway was complicated or predicted to be difficult, videolaryngoscopy using a C-MAC system (KARL STORZ, Tuttlingen, Germany) was used, offering greater visualization and the advantage of maintaining cervical spine stability.

For patients with fractures that precluded oral intubation or those requiring surgical access to the airway, blind nasal intubation was used. In such cases, the nasal cavity was adequately prepared using topical anesthetic agents, and an endotracheal tube was introduced through the nostril, guided by breath sounds and capnography. Throughout the procedure, all patients received appropriate analgesia, which included intravenous paracetamol and nalbuphine to ensure comfort while minimizing the risk of complications.

Anesthesia induction was achieved with propofol, and muscle relaxation was facilitated using atracurium. Isoflurane was used for maintenance of anesthesia, while the intubation technique was adjusted according to the patient's requirements. At the conclusion of the surgery, anesthesia was reversed using neostigmine and glycopyrrolate to ensure smooth extubation. Monitoring during the procedure was routine, with a difficult intubation cart on standby for any unforeseen challenges. All patients were extubated when fully awake and breathing spontaneously and responsive to commands.

Data were analyzed using IBM SPSS Statistics for Windows, Version 24 (Released 2016; IBM Corp., Armonk, New York). For age, we used mean with standard deviation, and for etiology of fractures, type of fractures, and airway management techniques, we used frequency and percentages. Data were presented in tabular form.

## Results

The study encompassed a retrospective analysis of 100 cases to evaluate airway management challenges in maxillofacial trauma presenting to the oral and maxillofacial emergency department. The cohort comprised patients aged 6 to 65 years, with a mean age of 33.33 ± 18.14 years. Males were in the majority, 65 (65.0%), while female patients made up 35 (35.0%) of the total.

Regarding trauma etiology, road traffic accidents were the most prevalent cause, 39 (39.0%), followed by falls from height, 36 (36.0%). Less frequent causes included interpersonal violence or gunshot injuries, 10 (10.0%), and sports-related incidents, 15 (15.0%). Fracture patterns varied, with mandibular fractures being the most common, 29 (29.0%), followed by nasal and maxillary fractures, 24 (24.0%) each, and orbital fractures, 23 (23.0%), as shown in Table 1.

**Table 1:** Clinical presentation of the patients

Clinical presentation	Category	Frequency	Percentage
Etiology of trauma	Road traffic accident	39	39.0%
	Fall from height	36	36.0%
	Interpersonal violence/gunshot	10	10.0%
	Sports	15	15.0%
Type of fracture	Nasal fracture	24	24.0%
	Mandibular fracture	29	29.0%
	Orbital fracture	23	23.0%
	Maxillary fracture	24	24.0%

Regarding the airway management strategies, direct laryngoscopy was performed in 53 (53.0%) patients, while videolaryngoscopy and blind nasal intubation were performed less frequently, i.e., 23 (23.0%) and 24 (24.0%), respectively, as shown in Table 2.

**Table 2:** Airway management techniques

Airway management	Frequency	Percentage
Direct laryngoscopy	53	53.0%
Videolaryngoscopy	23	23.0%
Blind nasal intubation	24	24.0%

## Discussion

Our retrospective analysis reveals that airway management poses significant challenges due to the difficult anatomy caused by fractures, tissue edema, hemorrhage, and potential cervical spine involvement. Our study revealed that road traffic accidents were the leading cause of trauma (39%), followed by falls (36%). The fractures observed most frequently in our patients were in the mandible, nasal area, maxilla, and orbital regions.

To secure the airway in these patients, we used a variety of techniques, with direct laryngoscopy being the most common management strategy. Other techniques, such as videolaryngoscopy and blind nasal intubation, were utilized less frequently. The choice of airway management strategy was heavily influenced by the type of fracture and the presence of associated complications, such as difficulty visualizing the larynx due to tissue swelling, blood, and debris.

These findings align with those in similar studies. For instance, in the study by Yang et al., certain types of traumatic facial injuries, such as Le Fort II fractures, bilateral mandibular fractures, and facial fractures with associated basilar skull fractures, were identified as key contributors to difficult intubation. Such injuries often lead to airway obstruction, requiring careful planning and execution of airway management techniques [12].

Similarly, Raval et al., in their study on airway management in maxillofacial trauma, revealed that nasal intubation with direct visualization was the most common method, while techniques such as oral intubation and fiberoptic bronchoscopic nasal intubation were used less frequently [13]. This finding is similar to our study, where methods such as videolaryngoscopy and blind nasal intubation were employed in a smaller proportion of cases, reflecting the variability in airway management approaches depending on the condition of the patient and the severity of the fracture.

In more severe cases, such as those with extensive facial trauma and possible airway obstruction, emergent procedures such as cricothyrotomy might be necessary. The study by Singh et al. emphasizes the need for such interventions when airway obstruction from soft tissue swelling or blood in the airway cannot be managed by less invasive techniques [14]. This is consistent with our findings, where videolaryngoscopy was considered in cases with airway obstruction, such as orbital fractures. In the cases of orbital fractures, videolaryngoscopy provides the added advantage of better visualization of the airway compared to other techniques, especially when there is distortion in the anatomy due to edema or blood accumulation.

Puolakkainen et al. highlight the significant role of timely airway management, particularly when facial fractures are accompanied by traumatic brain injuries (TBI) [15]. They found that patients with combined injuries often required on-scene airway management, emphasizing the importance of rapid assessment and intervention. This resonates with our findings, where airway management was critical for patients with complex facial fractures.

The clinical presentation of facial trauma patients often involves airway compromise, which requires a well-coordinated approach between anesthesiologists, maxillofacial surgeons, and other trauma specialists. As emphasized by Singh et al., airway management in these patients must be handled with particular care due to the risk of airway obstruction, bleeding, and soft tissue edema [14].

Our study highlights the complexities of airway management in maxillofacial trauma. While direct laryngoscopy, videolaryngoscopy, and nasal intubation are effective in many cases, each patient's injury profile must be considered carefully when selecting the appropriate technique.

Our study presents a few limitations. It relied on retrospective data, which may introduce selection bias and limit the ability to control for confounding variables. The absence of fiberoptic intubation due to its unavailability at the center restricts the range of techniques evaluated. Additionally, the lack of detailed reporting on patient outcomes, such as complication rates, narrows the scope of clinical insights provided.

## Conclusions

Our study concludes by emphasizing the intricacy and significance of efficient airway management in patients with maxillofacial trauma, highlighting the variety of strategies needed depending on the kind of fracture and related complications. The results highlight the necessity of specialized airway management techniques to guarantee optimal results in these demanding situations. To successfully manage airways and reduce problems, anesthesiologists, maxillofacial surgeons, and trauma specialists must work together.
